# YAP1 protects against septic liver injury via ferroptosis resistance

**DOI:** 10.1186/s13578-022-00902-7

**Published:** 2022-10-01

**Authors:** Jin Wang, Qian Zhu, Rui Li, Jing Zhang, Xujun Ye, Xinyi Li

**Affiliations:** 1 Department of Anesthesiology, Zhongnan Hospital of Wuhan University, Wuhan, Hubei China; 2 Department of Hepatobiliary and Pancreatic Surgery, Zhongnan Hospital of Wuhan University, Wuhan, Hubei China; 3 Department of Geriatrics, Zhongnan Hospital of Wuhan University, Wuhan, Hubei China

**Keywords:** Sepsis, Liver injury, YAP1, Ferritinophagy, Ferroptosis

## Abstract

**Background:**

The liver plays crucial roles in sepsis and is one of the major targets for sepsis-related injuries. Ferroptosis, a newly emerged form of lytic cell death, has been implicated in sepsis related organ failure. Yes-associated protein1 (YAP1), a key regulator of the Hippo signaling pathway, may be involved in ferroptosis development. This study aimed to elucidate the role of YAP1 in septic liver injury through regulating ferroptosis, especially ferritinophagy-mediated ferroptosis.

**Results:**

Cecal ligation and puncture (CLP) models were constructed in control (*Yap1*^*flfl*^) and liver-conditional knockout mice (*Yap1*^*fl/fl*^ Alb-Cre) to induce septic liver injury, while LO2 cells with or without YAP1 overexpression/deletion were stimulated by lipopolysaccharide (LPS) in vitro. Our study showed YAP1 knockdown aggravated CLP-induced liver injury and inflammation, as well as accelerated hepatocyte ferroptosis, revealed by down-regulated expression of GPX4, FTH1 and SLC7A11, along with up-regulated expression of SFXN1 and NCOA4. Consistently, YAP1 deficiency aggravated LO2 cells ferroptosis, but YAP1 overexpression alleviated LPS-induced LO2 ferritinophagy, as evidenced by reduced mitochondrial ROS and Fe^2+^, along with down-regulated expression of SFXN1 and NCOA4. Further co-IP assay verified that YAP1 disrupted the interaction between NCOA4 and FTH1, thus prevent the degradation of ferritin to Fe^2+^, further reduced the ROS production and suppressed ferroptosis.

**Conclusion:**

YAP1 inhibits ferritinophagy-mediated ferroptosis in hepatocytes, and YAP1 deficiency aggravates sepsis-induced liver injury.

**Supplementary Information:**

The online version contains supplementary material available at 10.1186/s13578-022-00902-7.

## Introduction

Sepsis is a kind of potentially life-threatening, systemic inflammatory response syndrome (SIRS) that results from the spread of pathogenic agents (such as bacteria or viruses) and their toxins to the bloodstream, which in severe cases can induce septic shock and multiple organ dysfunction syndrome (MODS) [1]. As a critical organ, liver plays a central role in metabolism and immune homeostasis, which are most frequently implicated by sepsis [2]. Clinical evidence indicates hepatic dysfunction is an early event in sepsis, and has been use as a strong predictor of high mortality and poor prognosis in critically ill patients [3–5]. The mechanisms of acute liver injury induced by septic shock include inflammation factors, oxidative stress, autophagy, cell apoptosis, and others [6, 7]. Therefore, exploring the potential mechanism is crucial for attenuating liver injury, restoring liver function and improving mortality rates in patients with sepsis.

Ferroptosis is a unique form of iron-dependent cell death, induced by free iron and reactive oxygen species (ROS), resulted from lipid peroxidation. Ferroptosis is mainly regulated by iron homeostasis and oxidative stress, and iron homeostasis is partly controlled by ferritin. Ferritin consists of ferritin light chain (FTL) and ferritin heavy chain 1 (FTH1), the latter is the primary iron-storage protein. Nuclear receptor coactivator 4 (NCOA4), a selective cargo receptor of ferritin, could combine with ferritin, and transport ferritin into lysosomes, and then activate ferritin degradation there. The form of NCOA4-depended ferritin autophagy was defined as ferritinophagy. The process of ferritinophagy includes the release of a large amount of ferrous iron (Fe^2+^) through Fenton reaction to generate hydroxyl radicals [8]. In the pathological states (sepsis, inflammation, etc.), elevated Fe^2+^ stimulates the accumulation of sideroflexin 1 (SFXN1) to locate in mitochondrial membrane, which subsequently transports Fe^2+^ into mitochondria, triggering the production of mitochondrial ROS and ferroptosis [9]. It has been reported that ferroptosis participates in pathogenesis of sepsis-induced cardiac injury and liver injury [10, 11]. Although mounting evidence have reported a link between ferroptosis and sepsis, there are sporadic studies on the involvement of ferritinophagy in sepsis-induced liver injury.

Yes-associated protein 1 (YAP1), along with its transcriptional co-activator TAZ, serve as the crucial downstream effectors in the Hippo pathway, which plays an essential role in cell proliferation and organ development. The Hippo/YAP1 pathway is a kinase cascade, and YAP1 can be directly phosphorylated and inactivated by large tumor suppressor 1/2 (LATS1/2), resulting in of YAP1 cytoplasm reservation [12]. Previous studies have reported the positive effect of YAP1 in cell proliferation and regeneration. Deng et al. found that nuclear YAP1 exerted an anti-apoptosis effects and drove intestinal epithelial cell proliferation and regeneration in ulcerative colitis [13]. A single-Cell Analysis revealed that YAP played an essential role in hepatocyte regeneration and is required for hepatocyte homeostasis [14]. Liu et al. reported that YAP1 activation promoted hepatocyte regeneration and attenuated hepatic damage under liver ischemia-reperfusion stress [15]. Accumulating evidence have supported the protective effect of YAP1 in liver disease, nevertheless, a small amount researches involved the YAP1 function in the genesis and development of septic liver injury. Besides, it has also been reported that YAP1 affects the production of autophagosomes and regulate autophagy, which is a crucial element of ferritinophagy [8, 16]. Since recent investigations have already explored the specific mechanism of YAP1 related to NCOA4-mediated ferritinophagy or FTH1 degradation, we hypothesized that YAP1 inhibited NCOA4-mediated ferritinophagy, producing a decrease in free ferrous iron, and protected hepatocyte from ferroptosis in sepsis mice.

## Materials and methods

### Animals and models

Male 8–12 weeks old wild-type (WT) mice (Cyagen, Suzhou, China) on the C57BL/6 background were used in this experiment. YAP1^flox/flox^ mice were crossbred with Alb-Cre mice to generate liver-specific YAP1-deficient mice (termed as YAP1^fl/fl^ Alb-Cre) [17]. Tamoxifen (Sanbio #13258) was intraperitoneally injected on five consecutive days (16 mg/kg in oil), followed by a 3-week wash out period. The control group mice were Yap^flox/flox^. Control mice and YAP1-deficient mice were randomly distributed into sham and CLP groups. All experiments were executed following the criteria of the NIH and authorized by Animal Ethics Committee of Wuhan University (No. 2021187).

According to previous researches [18], The sepsis-associated liver injury model mice were established by cecal ligation and puncture (CLP). After anaesthetized with an intraperitoneal injection of pentobarbital sodium (50 mg/kg), the abdomen was sterilized with povidone iodine, then about 2 cm midline incision was conducted in the mice’s lower abdomen. After that, the cecum was exposed and ligated with a 3-0 silk. We punctured the cecum with a 21-gauge needle twice. After gently squeezing the cecum to push the feces into the abdominal cavity, the cecum was put back to the original location followed by suturing the peritoneum layer by layer. Volume resuscitation was performed immediately after surgery with 30 ml/kg ringer saline. Only abdominal incision was performed on the WT mice, exposed for 5 min and then closed in layers. The mice enjoyed free access of food and water and were monitored until been sacrificed at 24 h post-operation.

### Liver function and pro-inflammatory factors assessment

Plasma levels of alanine aminotransferase (ALT) and aspartate aminotransferase (AST) were analyzed using the commercial ELISA kits (R&D Systems, USA) following the manufacturer’s instructions. TNF-α, IL-1β and IL-6 in liver tissue were detected using the Duoset ELISA system (R&D systems, MN) according to the manufacturer’s instructions.

### Transmission electron microscopy (TEM)

The left liver lobe was resected and fixed in glutaraldehyde, then the samples were dehydrated with a cascade ethanol series and cut into ultrathin slices. The slices were dyed with lead citrate and uranyl acetate respectively, in turn, observed using a HT-7500 transmission electron microscope (Hitachi. Co., Tokyo, Japan).

### Histological analysis

The left-liver samples were isolated and fixed with 4% paraformaldehyde. Following paraffin embedding and slicing, hematoxylin–eosin (HE), macrophages (CD68) (AbD Serotec, Raleigh, NC), neutrophils (Ly6G) (BD Biosciences, San Jose, CA), was utilized to stain liver samples. The degree of liver damage was assessed by two independent technicians who were unknown the experimental group protocols in line with the recently published criterion [19]. The histological photographs were observed by optical microscopy (Nikon, Japan).

### Immunohistochemical stain (IHC stain)

Liver tissues were paraffin fixed and incubated overnight at 37 °C. Then, these liver sections were deparaffinized and hatched with 3% hydrogen peroxide for 15 min. The slices were heated at microwave treatment and then naturally cooled for 40 min. Then the samples were incubated with anti-NCOA4 and anti-SLC7A11. Lastly, homologous fluorescent or biotin-labeled secondary antibodies were incubated with the liver tissues for observing protein expression.

### Primary hepatocytes culture and treatments

Mice were killed and perfused with 75% alcohol for 2–3 s. For the isolation of hepatocytes, livers were then taken and minced, then placed in a dish containing PBS. Then, the buffer was replaced with 0.1% collagenase I solution (Sigma, C0130) in HBSS (containing 4 mM CaCl_2_, 0.8 mM MgSO_4_) at 37 °C for 10 min. Freed from the liver, hepatocytes were collected and filtered through a sterile 100 µm pore size nylon mesh, then wash 2–3 times at 4 °C for 4 min. Hepatocytes were cultured in Williams’ E medium (Invitrogen, Carlsbad, CA) added with 100 lg/mL streptomycin, 10% fetal bovine serum, 100 U/mL penicillin, 0.5 IU/mL insulin, and 10 lg/mL dexamethasone. LPS (1 µg/mL, Sigma, Aldrich, USA) was given to generate cell injury models in vitro for 24 h.

### Cells transfection

Human liver LO2 cells (American Type Culture Collection [ATCC], Manassas, VA, USA, USA) were cultured with fetal bovine serum (10%) in DMEM (Gibco, USA) and arranged at an incubator containing 5% CO_2_ with suitable temperature (37 °C). YAP1 overexpression (YAP1 OE) clone lentiviral particle (supplied by Genechem Co. LTD Shanghai, China) were transfected to LO2 cells to constitute the stable YAP1 OE cell lines. Transfection of YAP1 was accomplished referring to the working instructions, cells transfected with scramble were used as negative controls. LO2 cell were transfected with siRNA specific for YAP1 (Shanghai GenePharma Co. China) by using Lipofectamine 2000 (Thermo Fisher Scientific, USA) according to the manufacturer’s instruction. Following expansion and maintenance, stable LO2 cells YAP1 OE or YAP1^−/−^ were selected to detection. LPS (1 µg/mL, Sigma, Aldrich, USA) was given to generate hepatocyte injury models in vitro for 24 h.

### Cell viability assay

As previously illustrated, cell viability was examined by the CCK-8 assay kit (Beyotim, Shanghai, China) [20].

### Determination of ROS

LO2 cells were suspended by 0.25% trypsin and subsequently centrifuged at 1500 rpm (4 °C, 5min).The cells were incubated with the 2′,7′-dichlorodihydrofluorescein diacetate (DCFH-DA) fluorescent probe (D6883, Sigma-Aldrich, MO, USA) in serum-free medium following the manufacturer’s protocols. The level of ROS in LO2 cells was acquired by a TE-2000 fluorescent microscope (Nikon, Tokyo, Japan) at an excitation (Ex) wavelength of 485 nm and emission (Em) wavelength of 530 nm. After BODIPY-581/591-C11 fluorescence probe was added into cell culture, a flow cytometry (BD Accuri C6 plus, BD Biosciences, USA) and FlowJo software were adopted aiming to detect the lipid ROS level.

The ROS level of liver tissues was evaluated with the dihydroethidium (DHE) fluorescent probe (D7008, Sigma-Aldrich, MO, USA). The already frozen sections were incubated with 50 μm DHE away from light at 37 °C for 30 min. To obtain the cell images, we used Nikon TE-2000 fluorescent microscope (Tokyo, Japan) at (Ex/Em) 525 nm/610 nm.

### Determination of mitochondria ROS

LO2 cells were seeded in 24-well lucifugal plate, then incubated with 5 mmol/L MitoSOX™ reagent solution for 10 min. The cells were washed two times by PBS, the level of mitochondria ROS was assessed by a fluorescence microscope (TE-2000, Nikon, Co., Tokyo, Japan).

### Detection of related indicators

The levels of ferrous iron (Fe^2+^), malondialdehyde (MDA) and glutathione (GSH) in LO2 cells and liver tissues lysate were detected by the iron assay kit (MAK025, Sigma-Aldrich, MO, USA), MDA assay kit (Beyotime, China) and GSH assay kit (Beyotime, Shanghai, China) according to the relevant manufacturer’s protocols.

### Western blot

We extracted the lysate from liver tissues or cells in a RIPA buffer and protein contents were quantified by the BCA protein assay kit (Beyotime, China). Protein samples (50 µg) from each group underwent the 10% SDS-PAGE gel electrophoresis and then transferred to a PVDF membrane. After blocked with nonfat dry milk (5%), these blots were hatched with the primary antibodies all night at 4 °C. GPX4, ACSL4, SFXN1, LC3, NCOA4, FTH1, SLC7A11, YAP1or β-actin. Moreover, the blots were cleaned in TBST and hatched with the secondary antibody at 37 °C lasting 120 min. An ECL kit was applied to detect the bands, which were assayed by the Image J software.

### Distribution of ferrous iron in the Mito Tracker

The MitoTracker Green fluorescent probe (Beyotime, China) was firstly added to the LO2 cells for 30 min, then the previous solution was replaced by fresh PBS. Subsequently, the prewarming Ferro Orange working solution was used to treat the cells kept in lucifuge place for 30 min, and PBS was used to wash the medium. The cell photographs were captured by a confocal microscope (TCS-SP2, Leica, German) to acquire the mitochondrial ferrous iron and green fluorescent lysosomes at Ex and Em wavelengths of 488 nm and 510–550 nm, respectively.

### Ferritin and LAMP2

LO2 Cells were fixed as pre-described and immunostained by anti-ferritin (Abcam, ab75973, UK) and anti-LAMP2 (Proteintech, 66301-1-Ig, USA). Then, samples were incubated with secondary antibodies or DAPI. LO2 cells were photographed using Confocal laser scanning microscopy and fluorescence microscopy (TE-2000, Nikon, Co., Tokyo, Japan). Quantification of the ferritin and lysosomes in each hole cells were analyzed and calculated by software Image-pro Plus 6.0.

### Co-localization of LC3 and ferritin

LO2 cells in 4 groups were transfected with LC3-GFP plasmid, while the autophagosome in cells presented green fluorescence puncta. Following the manipulation manuals, we firstly blocked the cells with 0.1% BSA (A2153, Sigma-Aldrich, USA) for 1 h, then applied the primary and secondary antibodies to the medium. After using anti-Ferritin Heavy chain1, LO2 cells were analyzed via Confocal laser scanning microscopy and evaluated by fluorescence microscopy (Nikon TE-2000, Tokyo, Japan).

### Co-immunoprecipitation (IP) assay

The concentration of protein in LO2 cells was assessed by BCA protein (Beyotime, Shanghai, China) assay kit. 1 mg total protein lysates extracted from each group were utilized for immunoprecipitation (IP). The specimen was hatched with rabbit polyclonal IgG control antibody. After rotating the lysates lasting 4 h at 4 °C, a total volume of 25 µL resuspended of protein A/G plus Agarose was mixed with the lysates and the mixture solution continued rotating lasting 2 h again. The immunoprecipitation buffer was washed and denatured, then the elution proteins were immunoblotted with the antibody (anti-NCOA4, anti-Ferritin), the co-IP assay was performed.

### RNA sequence analysis

First, RNA was extracted from CLP or normal liver tissue. Bioanalyzer 2100 system was used to detect RNA purity. Ribo-off rRNA Depletion Kit (N406-02, Vazyme, China) and MGIEasy RNA Library Prep Kit (1000006385, MGI, China) were used as to generate sequencing libraries in terms of manufacturer’s instructions. Then, after purifying the PCR products by the AMPureXP system, the library quality was analyzed (Agilent Bioanalyzer 2100 system). Finally, the index codes were clustered by Cluster Kit of HiSeq 4000 PE (Illumina, USA), the sequenced library preparations were performed for 150 cycles on the former platform. Total library constructions and sequencing were implemented at BGI Wuhan. Hierarchical clustered heatmap and Genomes enrichment analysis were adopted to detect the differential expression of both groups. Differentially expressed genes (DEGs) were displayed for log2 Fold Change > 1 and adjusted p value < 0.05.

### Statistical analysis

The Prism 8.0 software (GraphPad, San Diego, CA) was used to analyze the data presented as Means ± SD. Comparisons between two groups were performed with a two-tailed unpaired t-test. Discrepancies among four groups were checked by one-way ANOVA analysis. Welch correction was applied when necessary. *P* < 0.05 was defined as statistically significant.

## Results

### YAP1 deficiency aggravated oxidative stress-mediated liver injury in CLP mice

**Fig. 1:**
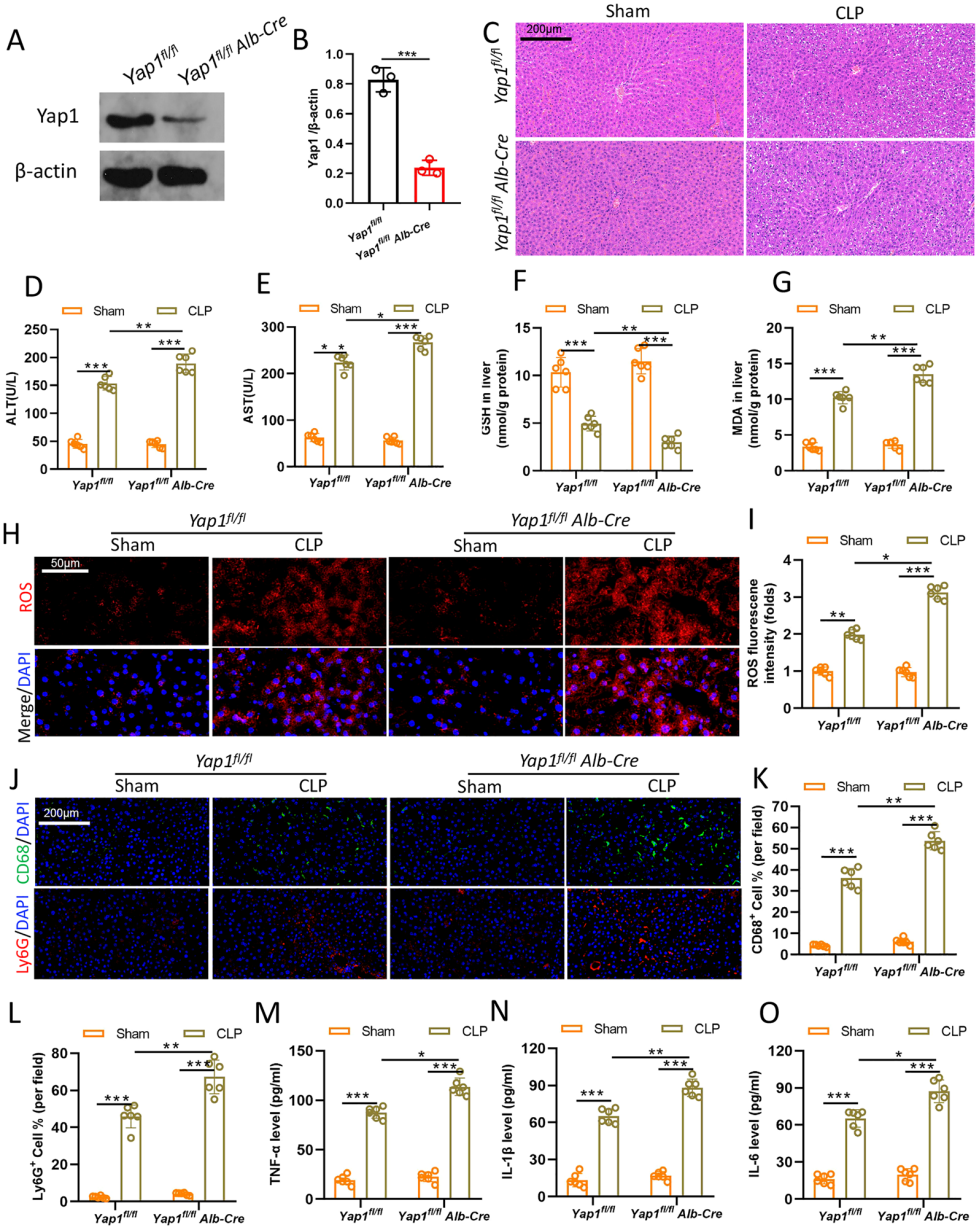
YAP1 deficiency aggravated oxidative stress-mediated liver injury in CLP mice. 8–10-week-old control (YAP1^flfl^ mice) and YAP1^fl/fl^ Alb-Cre mice were randomly distributed into sham and CLP groups. **A**, **B** The certification of YAP1 conditional knockout in mice liver tissues, n = 3. **C** Histological images of liver samples exhibited by HE staining in YAP1^fl/fl^ mice or YAP1^fl/fl^ Alb-Cre mice with or without CLP treatment. **D**, **E** ALT and AST in liver tissue, n = 6. **F**, **G** Representative fluorescent images of ROS staining and the quantification of ROS fluorescence intensity in liver, n = 6. Liver content of GSH (**H**) and MDA (**I**) assessed by corresponding commercial kit, n = 6. **J**, **L** Representative images of the inflammatory cells infiltration as reflected by immunohistochemistry staining for CD68 and Ly6G in live and the quantification of fluorescence intensity, n = 6. **M**–**O **The levels of TNF-α, IL-1β and IL-6 in liver tissues of each group, n = 6. Data are expressed as mean ± SD of three replicates. *p < 0.05, **p < 0.01, ***p < 0.001

To verify the involvement of YAP1 in sepsis-induced liver injury, we used the YAP1 liver-conditional knockout mice. As shown in Fig. 1A, B western bolt analysis confirmed that YAP1 protein remarkedly decreased in YAP1^fl/lfl^ Alb-Cre liver tissue, indicating that the CKO-model was successfully established. As shown in Fig. 1C, CLP produced significantly liver damage including inflammatory infiltration, disordered arrangement, hepatocyte swelling, and cell necrosis, while mice lacking YAP1 exhibit more severe pathological alterations. Besides, YAP1 deletion exacerbated liver cell injury significantly as evidenced by elevating ALT and AST in liver tissue (Fig. 1D, E). To further demonstrate the protection of YAP1 against oxidative stress in CLP-treated mice, we measured the ROS production, MDA, and GSH with or without YAP1 deficiency in the murine liver tissues. CLP-stimuli markedly aggrandized the generation of ROS and MDA levels and diminished GSH production, while YAP1 deficiency exacerbated the oxidative damage caused by CLP (Fig. 1F–I). Inflammatory response activation is one of the most important pathological alterations in septic liver. Therefore, we next investigated immune response and levels of pro-inflammatory cytokines in each group. The infiltration of Ly6G-labeled neutrophils and CD68-labeled macrophages caused by LPS was aggravated in YAP1 deficiency (Fig. 1J, K). Similarly, YAP1 inhibition promoted inflammation, as evidenced by the increased levels of TNF-α, IL-1β, IL-6 in liver tissue (Fig. 1M–O). Collectively, we considered that YAP1 knockdown aggravated oxidative stress-mediated liver injury in CLP mice.

### YAP1 deficiency aggravated CLP-induced ferroptosis and ferritinophagy in liver tissues

**Fig. 2:**
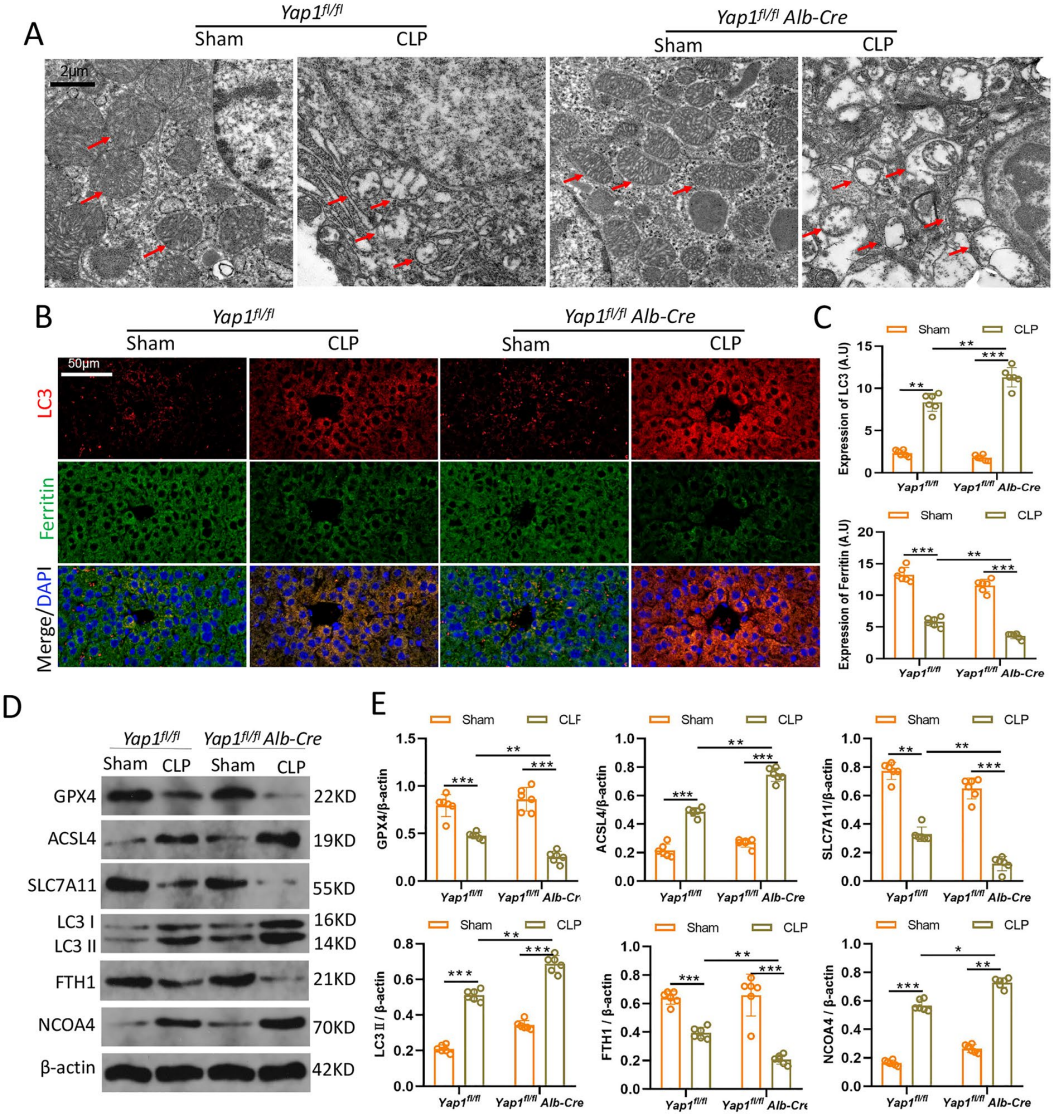
YAP1 deficiency aggravated CLP-induced ferroptosis and ferritinophagy in liver tissues. **A** Representative images showed by TEM. The red arrow indicates representative mitochondria in YAP1^fl/fl^ or YAP1^fl/fl^ Alb-Cre mice liver treated by CLP or not. **B**, **C** Fluorescent analysis showed representative images of colocalization ferritin (green) with tissues expressed LC3 (red) and quantification fluorescence intensity of LC3, n = 6. **D**, **E** Immunobloting detection of the expression contents of GPX4, ACSL4, SLC7A11, LC3 (I, II), FTH1, and NCOA4 in mice with indicated treatment, n = 6. Data are expressed as mean ± SD of three replicates. *p < 0.05, **p < 0.01, ***p < 0.001

Ferroptosis is distinguished by changes in mitochondrial morphology. The morphological of mitochondrial were captured with TEM to investigate whether aggravated liver injury was mediated through ferroptosis. The photographs revealed CLP caused significant aberrant mitochondrial (red arrows), including reduction even disappearance of mitochondria cristae and rupture of mitochondrial outer membrane. YAP1 deficiency exacerbated CLP-induced mitochondrial damages (Fig. 2A). Furthermore, to explore the effects of YAP1 on ferritinophagy, we used immunofluorescence staining to determine the colocalization of LC3 (biomaker of autophagosome, red) and ferritin (cytosolic iron storage complex, green). CLP treatment triggered the autophagy and promoted the degradation of ferritin in liver tissue, while YAP1 deficiency amplified these effects (Fig. 2B, C), indicating that YAP1 knockdown could promote ferritinophagy induced by CLP in liver. Known as ferritinophagy, NCOA4 is a specific cargo receptor that mediates the autophagic degradation of ferritin. Consequently, the expression of LC3II, ACSL4, and NCOA4 were significantly increased, while GPX4, SLA7A11 and FTH1 was sharply decreased after CLP compared with the control mice. In YAP1 conditional knockout mice, LC3II, ACSL4 and NCOA4 climbed to more higher levels, while GPX4, SLA7A11 and FTH1 presented massive degradation (Fig. 2D, E). In summary, our results suggested that YAP1 deficiency exacerbated ferroptosis and ferritinophagy in CLP mice.

### RNA-seq identified the enriched ferroptosis pathway in sepsis-induced liver injury

**Fig. 3:**
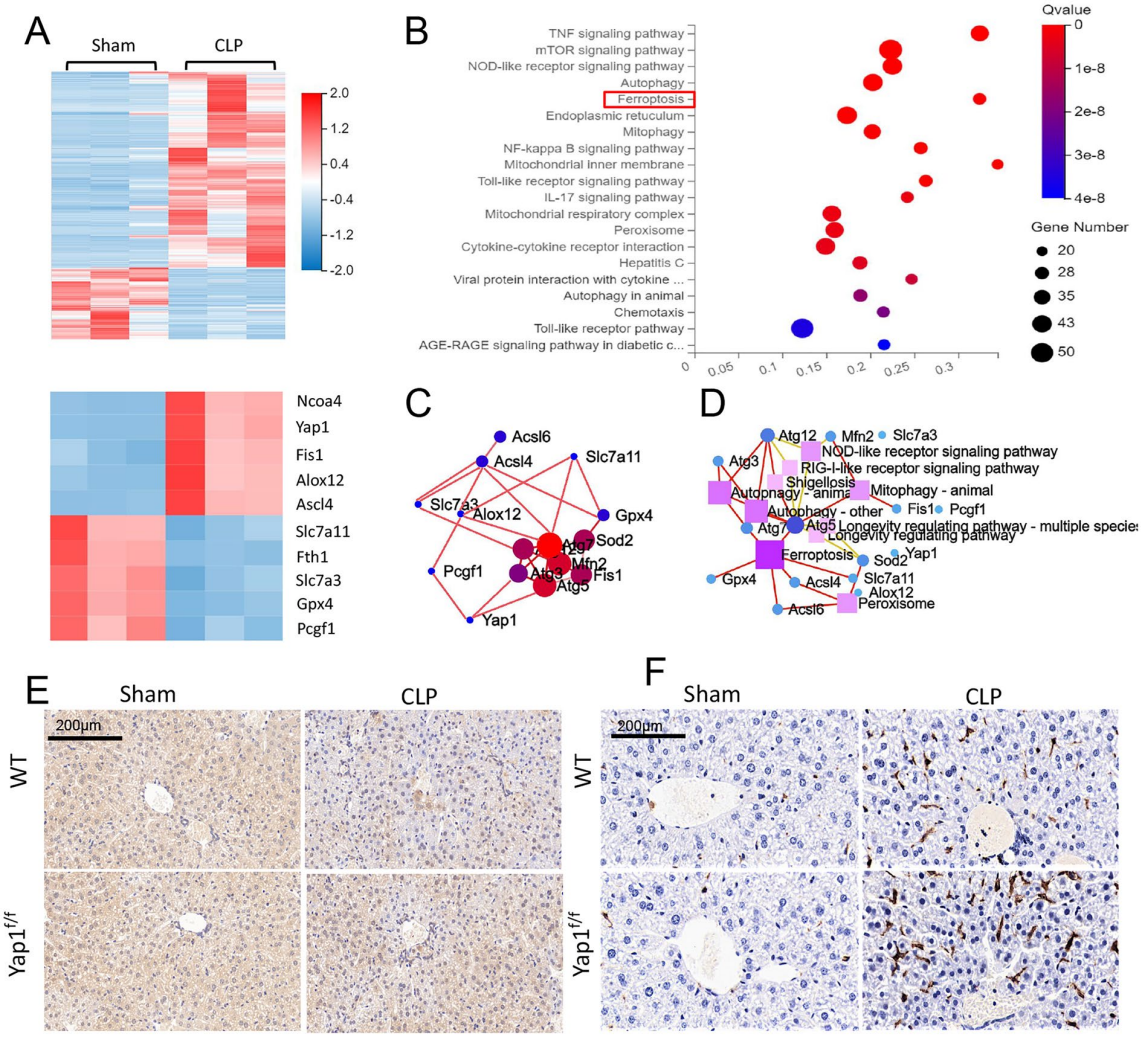
Gene expression analysis of liver tissue in CLP mice and WT mice. **A** Hierarchical clustered heatmap of differentially expressed genes (DEGs) in liver tissues between CLP mice and WT mice, n = 3 log2 FC > 1, Q value < 0.05. **B** Kyoto Encyclopedia of Genes and Genomes enrichment analysis(KEGG pathway)was adopted to identify the most significantly altered signaling pathways in liver tissues of CLP mice, n = 3. **C** Construction of protein-protein interaction (PPI) regulatory network based on DEGs. **D**, **E** Network of KEGG pathway based on similarity of their gene expression profiles. **F**, **G** SLC7A11 and NCOA4 of mouse liver tissues was stained by Immunohistochemical

To quantify the gene expression profiles of CLP treatment, RNA sequencing (RNA-seq) was performed in liver tissues of CLP mice or sham group. A hierarchical clustered heatmap identified a series of up-regulated genes (ALOX12, NCOA4, Fis1, YAP1) linked to ferroptosis and mitochondria fission as well as mitigation of genes (GPX4, Slc7a11, FTH1) associated with anti-oxidative stress and anti-ferroptosis (Fig. 3A). Genomes (KEGG) enrichment analysis and Kyoto Encyclopedia of Genes was utilized to identify the most significantly altered signaling pathways, including NF-κB signaling pathway and ferroptosis signaling pathway (Fig. 3B). The protein-protein interaction (PPI) regulatory network analysis predicted a high clustering coefficient and potential interaction among several related genes (Fig. 3C). Network of KEGG pathway elucidated a potential crosstalk of different signaling pathways (Fig. 3D, E). YAP1 promoted ferroptosis in CLP mice liver tissues, according to RNA-seq results, which gave us a hint to further investigate the molecular mechanisms and the relationship between YAP1 and ferroptosis. Moreover, as shown in immunohistochemical staining (Fig. 3F, G), SLC7A11 (brown dye) was accumulated in lung tissue but was reduced by CLP stimulation and further decreased by YAP1 knockdown. Additionally, we noticed that CLP stimulation caused NCOA4 (brown dye) to accumulate, and YAP1 deficient mice had more NCOA4 accumulation. These findings indicate that ferroptosis is induced during CLP treatment, and YAP1 plays a role in the regulating process.

### YAP1 knockdown promoted LPS-induced ROS accumulation and lipid peroxidation in vitro

**Fig. 4:**
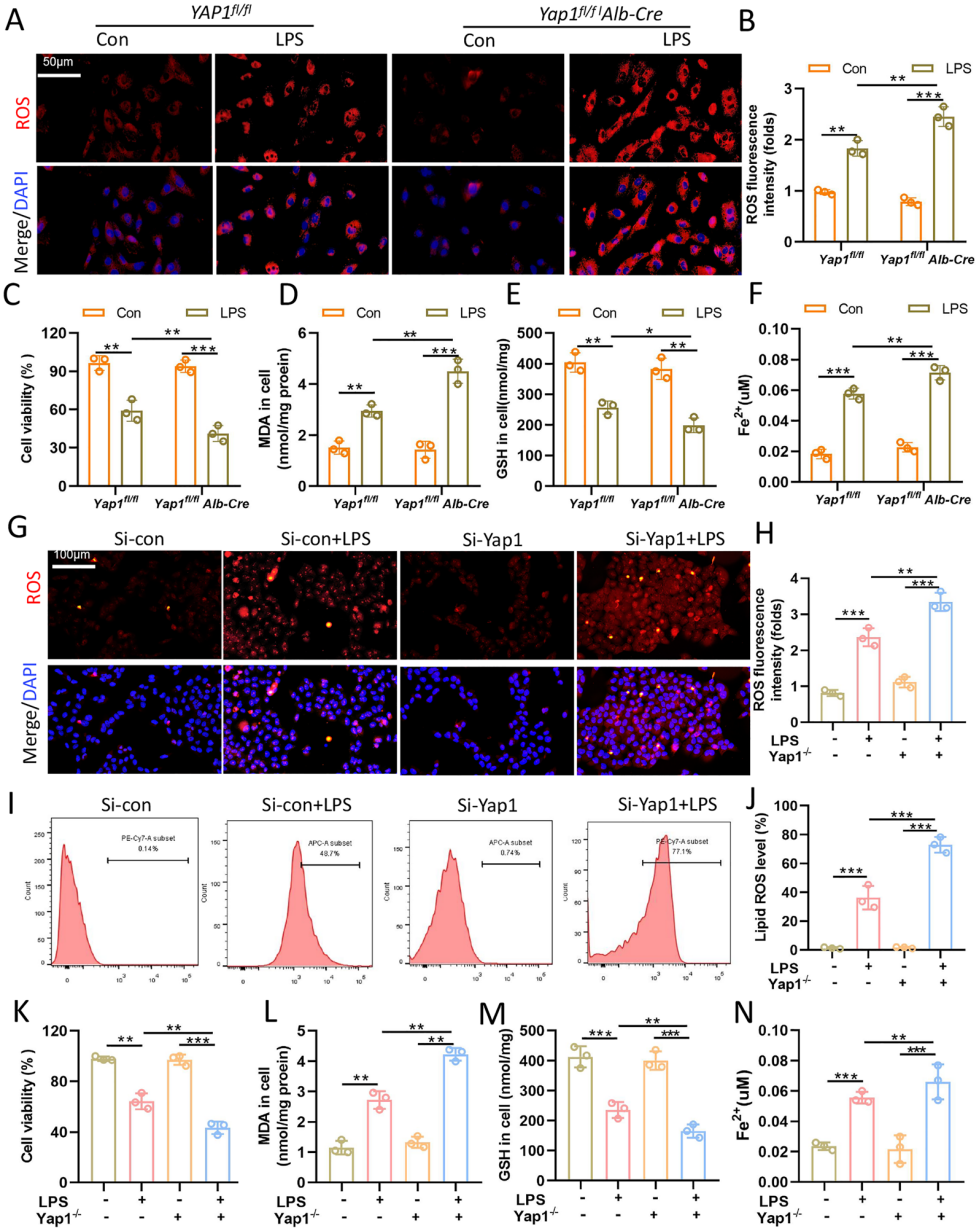
Yap1 deletion promoted LPS-induced accumulation of ROS and lipid peroxidation in vitro. Primary hepatocytes from YAP1^fl/fl^ and YAP1^fl/fl^ Alb-Cre mice and LO2 cells were treated with LPS (1 µg/mL) for 24 h. Representative images of fluorescence probes (DCFH-DA) for intracellular ROS production of primary hepatocytes (**A**) and LO2 cells (**G)** and the quantification of ROS fluorescence intensity respectively, n = 3 (**B**, **H**). Cell viability was evaluated by the cell counting kit-8 of primary hepatocytes (**C**) and LO2 cells (**K**) respectively, n = 3. The contents of MDA (**D**, **L**), GSH (**E**, **M**) and Fe^2+ ^(**F**, **N**) of primary hepatocytes and LO2 cells were determined using the indicated kits, n = 3. **I** Lipid ROS generation was analyzed by using BODIPY 581/591 and determined by flow cytometry in LO2 cells treated with LPS and **J** quantification of Lipid ROS, n = 3. Data are expressed as mean ± SD of three replicates, *p < 0.05, **p < 0.01, ***p < 0.001

Ferroptosis is a type of regulated cell death (RCD) accompanied by redox-active iron accumulation, GSH (glutathione) depletion, and lipid peroxidation [21]. In vivo, we observed that YAP1 appeared to play a regulatory role in CLP-induced ferroptosis, and we would like to explore the molecular mechanism further in vitro*.* Cell were treated with LPS (1 µg/mL) for 24 h to establish the cell model of sepsis (Additional file 1: Fig. S1). After that, in order to determine whether LPS caused the ferroptosis of LO2 cells, we evaluated the effects of the ferroptosis inducer (erastin) and inhibitor (Fer-1) on LPS-treated cells. As a result, LPS significantly increased iron and ROS production and decreased the GSH level in LO2 cells, while Fer-1 could reduce the iron accumulation, cut down ROS generation, but elevate GSH. Erastin had negative effects (Additional file 1: Fig. S2). These findings demonstrated that LPS stimulation of LO2 cells resulted in the activation of ferroptosis. To further explore the role of YAP1 in regulating ferroptosis, we conducted in vitro experiments with both primary hepatocytes from YAP1^fl/fl^ and Yap1^fl/fl^ Alb-Cre mice and LO2 cells transfected with YAP siRNA with LPS-stimulation. As we expected, ferroptotic events, including ROS (Fig. 4A, B, G, H) and lipid ROS generation (Fig. 4I, J), MDA production (Fig. 4D, L), GSH depletion (Fig. 4E, M), Fe^2+^ accumulation (Fig. 4F, N) were significantly triggered in both the two cell models, while YAP1 deficiency augmented these effects. Besides, LPS significantly reduced the cell viability in both the two cell models, which was further decreased after YAP1 inhibition (Fig. 4C, K). Collectively, these findings suggested that YAP1 knockdown promoted LPS-induced ROS accumulation and aggravated oxidative injury in vitro.

### YAP1 restrained SFXN1-mediated mitochondria transportation of Fe^2+^

**Fig. 5:**
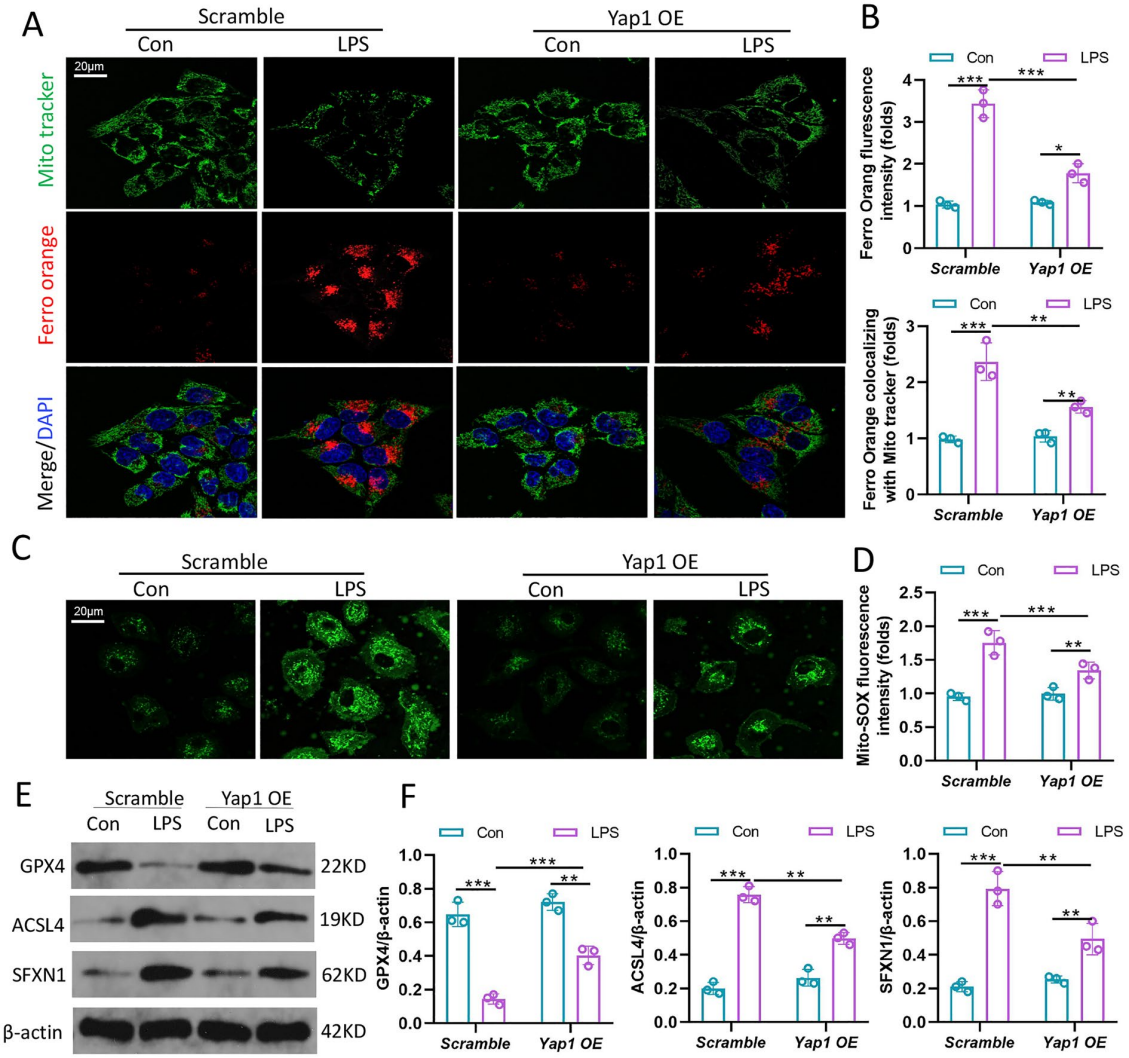
YAP1 activation inhibited ferroptosis by decreasing the amount of intracellular free ferrous iron that accumulated in mitochondria. LO2 cells transfected with YAP1 overexpression (YAP1 OE) clone lentiviral particle to constitute YAP1 OE cell lines. **A** MitoTracker (green) and Ferro Orange (red) were showed by co-staining in LO2 cells stimulated with LPS. Ferro Orange (red) exhibited the localization of intracellular free iron in living cells. The mitochondria were stained by Mito Tracker Green fluorescence. **B** Quantification of the fluorescence intensity of Ferro Orange colocalized with MitoTracker (green) and Ferro Orange fluorescence intensity, n = 3. **C**, **D** Representative images of Mitochondrial ROS stained by Mito-SOX and Quantitative results of Mitochondrial ROS, n = 3. **E**, **F** The expression levels of GPX4, ACSL4 and SFXN1 were showed by WB, n = 3. Data are expressed as mean ± SD of three replicates. *p < 0.05, **p < 0.01, ***p < 0.001

Intracellular iron overload induced the production of mitochondrial ROS and ferroptosis, which was mediated by the mitochondrial membrane protein SFXN1. To further explore the regulatory role of YAP1 in iron homeostasis, we transfected LO2 cells with YAP1 overexpression (YAP1 OE) clone lentiviral particle to constitute the stable YAP1 OE cell lines. First, We detected activation state of YAP1 by measuring the phosphorylation of YAP1 as well as intracellular location. The p-YAP1/YAP1 ratio in the liver dropped after LPS stimulation, indicating that YAP1 was activated. Moreover, YAP1 overexpression decreased the p-YAP1/YAP1 ratio, indicating that YAP1 was activated further (Additional file 1: Fig. S3A, B). Similarly, immunofluorescence studies revealed that LPS enhanced partial nuclei translocation of YAP1, while Yap1 overexpression in the presence of LPS considerably accelerated YAP1 nuclei translocation (Additional file 1: Fig. S3C). Then, we used the Fe^2+^-specific probe FerroOrange to determine if YAP1 could affect the concentration of bioavailable Fe^2+^. After LPS stimulation, the red fluorescence of Fe^2+^ increased, while the green fluorescence of mitochondria decreased, as well as the colocalization of Mito Tracker (green) and FerroOrange (red) strengthened, indicating that significant levels of iron were accumulated in mitochondrial (Fig. 5A, B). However, the distribution of free Fe^2+^ in mitochondrial was partially reduced when YAP1 was overexpressed (Fig. 5A, B). Using Mito-SOX green fluorescence probe, We observed that mitochondria ROS remarkably increased in LPS-induced cells. However, when YAP1 was activated, mitochondrial ROS exhibited a significant reduction trend (Fig. 5C, D). GPX4, which is one of mitochondria defense mechanisms, was inhibited after LPS-stimuli, but elevated after YAP1 activation (Fig. 5E, F). As demonstrated, YAP1 overexpression not only reduced the level of ACSL4, but also hindered the expression of SFXN1 after LPS induction (Fig. 5E, F). To some extent, YAP1 inhibited SFXN1-mediated deposition of intracellular free iron in mitochondrial and generation of mitochondrial ROS.

### YAP1 blocked the degradation of Ferritin in Lysosomes via inhibition of ferritinophagy

**Fig. 6:**
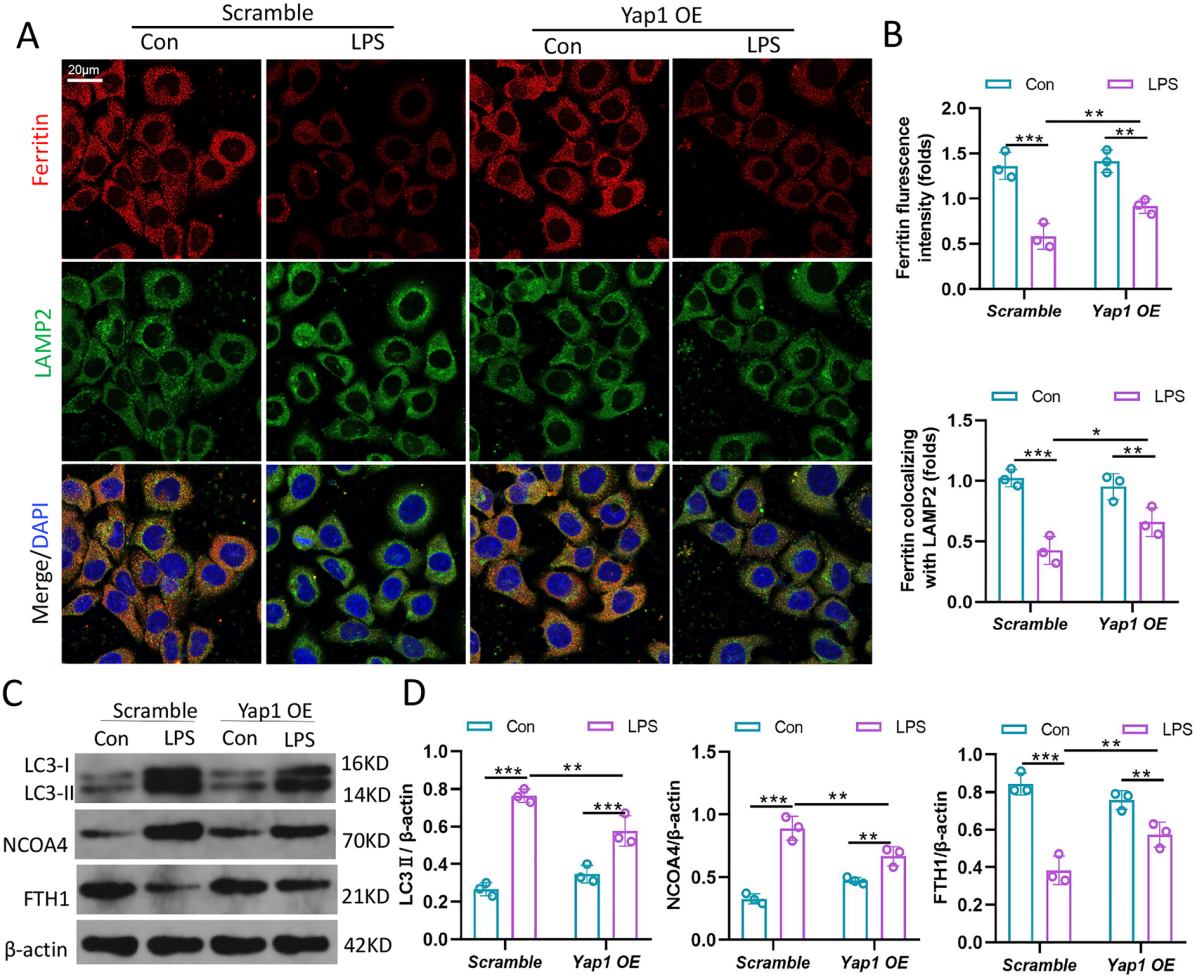
YAP1 restrained the degradation of ferritin in lysosomes to suppress ferritinophagy. **A** Confocal images of ferritin (red) and LAMP2 (green) were showed in LPS treated LO2 cells, while lysosomes were stained by LAMP2 (green). **B** Quantification measurement of the immunofluorescence intensity of ferritin colocalized with LAMP2 and ferritin fluorescence intensity, n = 3. **C**, **D** Data showed the expression contents of LC3 (II, I), NCOA4 and FTH1, n = 3. Data are expressed as mean ± SD of three replicates., *p < 0.05, **p < 0.01, ***p < 0.001

Ferritin, the primary iron-storage protein in mammals, is essential for iron homeostasis and preventing Fenton-reaction. Since one source of the Fe^2+^ releasing to cytoplasm is the degradation of ferritin, and above data showed that YAP1 inhibits the accumulation of Fe^2+^ in mitochondrial, therefore, we speculated that YAP1 could suppress ferritinophagy. To test the hypothesis we carried out immunofluorescence assays to survey the colocation of ferritin with lysosome. The LO2 cells were immune-stained with ferritin antibodies (red fluorescence) and LAMP2 (lysosomes labeled, green fluorescence) antibodies, and the results showed that the YAP1 OE in LPS-induced cells caused more extensive ferritin fluorescence staining in comparison to scramble group (Fig. 6A, B). Through the observation to the location of punctate ferritin lysosomes, we demonstrated that YAP1 inhibited ferritin-lysosomes colocalization and impaired ferritinophagy after LPS-stimulation (Fig. 6A, B).

As a cargo receptor protein in the process of ferritinophagy, NCOA4 could bind to ferritin and transport ferritin into lysosome, contributing to the degradation of ferritin to release free iron [22]. Blocking autophagy or knocking out NCOA4 inhibits the labile accumulation of iron and ROS associated with ferroptosis thus prevents iron-related cell death [23]. To further investigate the ferritinophagy, we measured the levels of LC3, NCOA4 and FTH1 by western blotting. We found that LPS treatment enhanced the expression of autophagic marker LC3 and NCOA4 in LO2 cells, whereas YAP1 overexpression caused a decline of LC3 and NCOA4 expression, (Fig. 6C, D). In addition to fluorescence images, YAP1 overexpression reversed the decrease in FTH1 level caused by LPS treatment (Fig. 6C, D). Overall, YAP1 inhibited ferritinophagy in cells exposed to LPS.

### YAP1 inhibited ferritinophagy by disrupting the NCOA4–FTH1 interaction

**Fig. 7:**
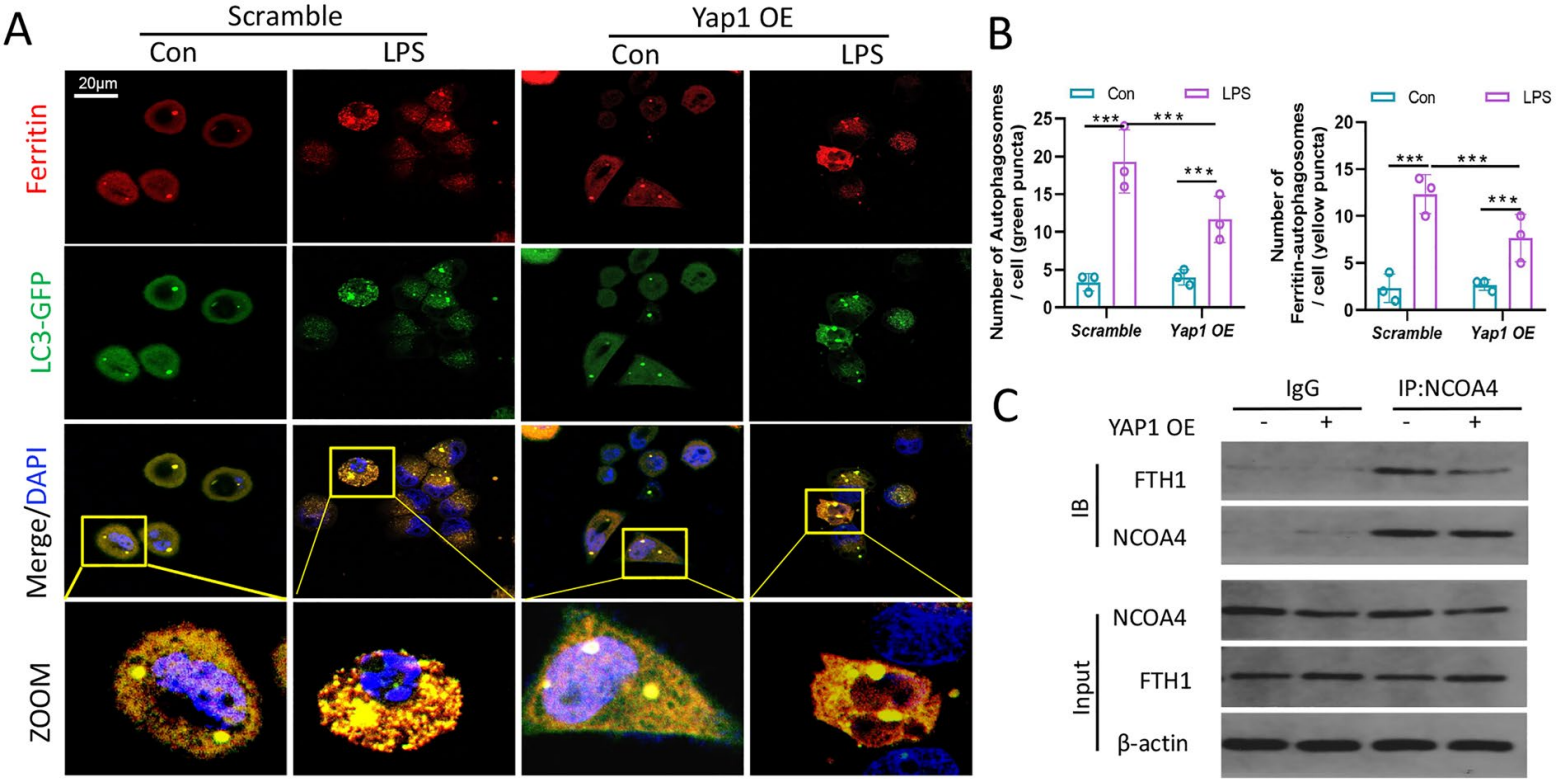
YAP1 inhibited ferritinophagy by disrupting the NCOA4–FTH1 interaction. **A** LO2 cells in 4 groups were transfected with LC3-GFP plasmid, while the autophagosome in cells presented green fluorescence puncta. Representative confocal images of cells exhibited fluorescent colocalization (yellow puncta) of ferritin (red fluorescence) with LC3-GFP (green puncta). **B** Fluorescence quantification of autophagosomes (LC3-GFP) and ferritin colocalizing with autophagosomes, n = 3. **C** Immunoprecipitation analysis of NCOA4 and FTH1 interaction in LO2 cells. IgG was used for control. Data are expressed as mean ± SD of three replicates, *p < 0.05, **p < 0.01, ***p < 0.001

Given the promoting role of autophagic ferritin degradation in ferroptosis, we preferred to elaborate the regulatory mechanisms for Fe^2+^ accumulation linked to ferroptosis generation in septic liver injury pathogenesis. The immunofluorescence analysis showed that several brilliant green spots emerged, demonstrating that LC3I was converted into LC3II, resulting in the formation of autophagosomes and the existence of autophagy. The expression of the ferritin was shown to be up-regulated by enhanced red fluorescent spots, whilst the colocalization of the ferritin and LC3 and the occurrence of ferritinophagy were shown by enhanced yellow fluorescent spots. However, YAP1 overexpression reduced the number of endogenous LC3 puncta as well as colocalization of ferritin with LC3 in the LPS-treated cells, demonstrating that YAP1 inhibited ferritinophagy (Fig. 7A, B). To further examine the relationship between of NCOA4-mediated ferritinophagy and FTH1, we performed co-IP assays to determine whether YAP1 suppressed the NCOA4–FTH1 interaction to prevent ferritinophagy. The immunoprecipitation analysis indicated that YAP1 overexpression decreased the amount of FTH1 bound with NCOA4, suggesting that YAP1 could disrupt the interaction between NCOA4 and FTH1 (Fig. 7C). Taken together, these outcomes hinted that YAP1 inhibited ferritinophagy by disrupting the NCOA4–FTH1 interaction.

## Discussion

This study focused on the potential role of YAP1 in ferroptosis during sepsis. The significant findings of the study are as follows: (1) YAP1 deficiency aggravated CLP-induced liver ferroptosis. (2) YAP1 disrupted the interaction between NCOA4 and FTH1, prevented the degradation of ferritin (ferritinophagy), thus reduced ROS generation and suppressed ferroptosis.

As an important transcription factor of Hippo pathway, YAP1 is regarded as a new light for the onset mechanism of various diseases, such as cancer, atherosclerosis, fibrosis, and inflammation. YAP1 has also been proved to promote cell proliferation and regeneration in some researches. YAP 1 could abolish inflammation and regulate the pulmonary endothelial cells activation through preventing TRAF6-mediated NF-κB activation [24]. In acute lung injury, YAP/TAZ deficiency in AECIIs exhibited long-term inflammatory responses and inhibited the epithelial regeneration [25]. Hence, given the protective role of YAP1 in promoting cell proliferation, we hypothesized that YAP1 exerted a protective effect in sepsis-induced liver injury. Our findings showed that YAP1 conditional knockout remarkably aggravated CLP-induced pathological change and inflammatory response in liver tissue. However, this mechanism requires further investigation, thus we explored the protective effect of YAP1 on ferroptosis involved in sepsis.

Ferroptosis is a novel form of regulated cell death, which is described as an iron-dependent cell death in consequence of phospholipid peroxidation induced by ROS production from iron-mediated Fenton reaction [26]. Ferroptosis is characterized morphologically by mitochondrial shrinkage and increased mitochondrial membrane density. Amino acid metabolism is closely related to ferroptosis. SLC7A11, GSH and GPX4 are pivotal regulators of ferroptosis pathway [27]. The cystine-glutamate reverse transporter System Xc—intakes cysteine, facilitating the synthesis of GSH and GPX4 antioxidant activity, thereby inhibiting ferroptosis [28]. GPX4 and GSH levels reduced during ferroptosis, resulting in lipid peroxidation [27, 29]. ACSL4 is also required for ferroptosis by inserting unsaturated arachidonic acid into the cellular membrane, which generates a large amount of lipid ROS in this process. Pharmacological inhibition of ACSL4 can prevent ferroptosis-related diseases [30]. Although growing evidence indicated that ferroptosis played a role in the genesis and progression of septic liver injury [31], there was no report about YAP1 function on ferroptosis in septic liver injury. In the current study, we noted that YAP1 conditional knockout aggravated sepsis-induced ferroptosis-like morphological changes, and produced more of ROS, MDA and Fe^2+^ production in liver tissues. Consistently, in vitro experiments demonstrated that YAP1 overexpression could decreased Fe^2+^ and lipid ROS accumulation. Moreover, our study also showed that YAP1 promoted the expression of SLC7A11 and increased the GPX4 and GSH level both in septic liver injury mice and LPS-stimulated LO2 cells, while suppressed the expression of ACSL4. These findings indicate that YAP1 suppresses ferroptosis via modulating Fe^2+^ and lipid peroxidation.

To explore the underlying mechanism of ferroptosis, RNA-seq was performed to identify the up-stream signal pathway. The PPI and KEGG network revealed a crosstalk between ferroptosis and autophagy, as evidenced by enriched ferroptosis and autophagy related genes and signal pathways (NCOA4, ACSL4, Atg3, Atg5, Atg12), prompting us to further investigate the mechanism of ferroptosis. Previous researches indicated that inhibiting autophagy contributed to a remarkable detente of ferroptosis, indicating that it is most probably reflective of an autophagic related cell death process [32, 33]. The process of autophagy involves continuous degradation of substrates in lysosomes for maintaining cell homeostasis in response to hypoxia, stress as well as sepsis. In the course of ferroptosis, autophagy is activated, contributing to the degradation of ferritin. NCOA4 mediates the autophagic degradation of ferritin to lysosomes (Ferritinophagy) via binding to ferritin, resulting a large amount of cellular iron release, which initiates Fenton reaction to transform free iron into Fe^2+^ [34]. These cascades of reactions could be blocked by NCOA4 inhibition, thus abrogating the accumulation of ferrous iron and ROS from ferroptosis [35]. In our experiments, the colocalization of ferritin and autophagosomes was observed by confocal microscopic evaluation, indicating that YAP1 restrained the degradation of ferritin in lysosomes to suppress ferritinophagy. Qi et al. clarified that curcumol could inhibit NCOA4 regulation of ferritinophagy to prevent hepatocyte senescence through up-regulation of YAP in fatty liver disease [36]. Zhang et al. revealed that YAP might inhibit ferroptosis via increasing FTH1 in level [37]. In this study, we discovered that YAP1 could also disrupt the interaction of NCOA4 and FTH1 by co-immunoprecipitation assays, which was consistent with the previous researches.

SFXN1 is closely related to pathologic mitochondrial iron accumulation in previous studies [38]. As a multifunctional mitochondrial membrane protein, SFXN1 not only transports iron to mitochondria to promote the utilization of erythroid mitochondrial iron, but also acted as mitochondrial serine transporter [39]. SFXN1-induced mitochondria iron accumulation is activated by NCOA4-mediated ferritinophagy under stress [39]. Increased mitochondrial ROS could trigger ferritinophagy and augment the content of intracellular iron, eventually leading to ferroptosis [40]. Our results indicated that YAP1 could suppress autophagy and degradation of Ferritin (located in lysosome), and inhibit the expression of SFXN1, eventually prevented a large amount of Fe^2+^ release and mitochondrial ROS generation, which was crucial for protecting hepatocyte cells from sepsis-induced ferroptosis.

## Conclusion

**Fig. 8:**
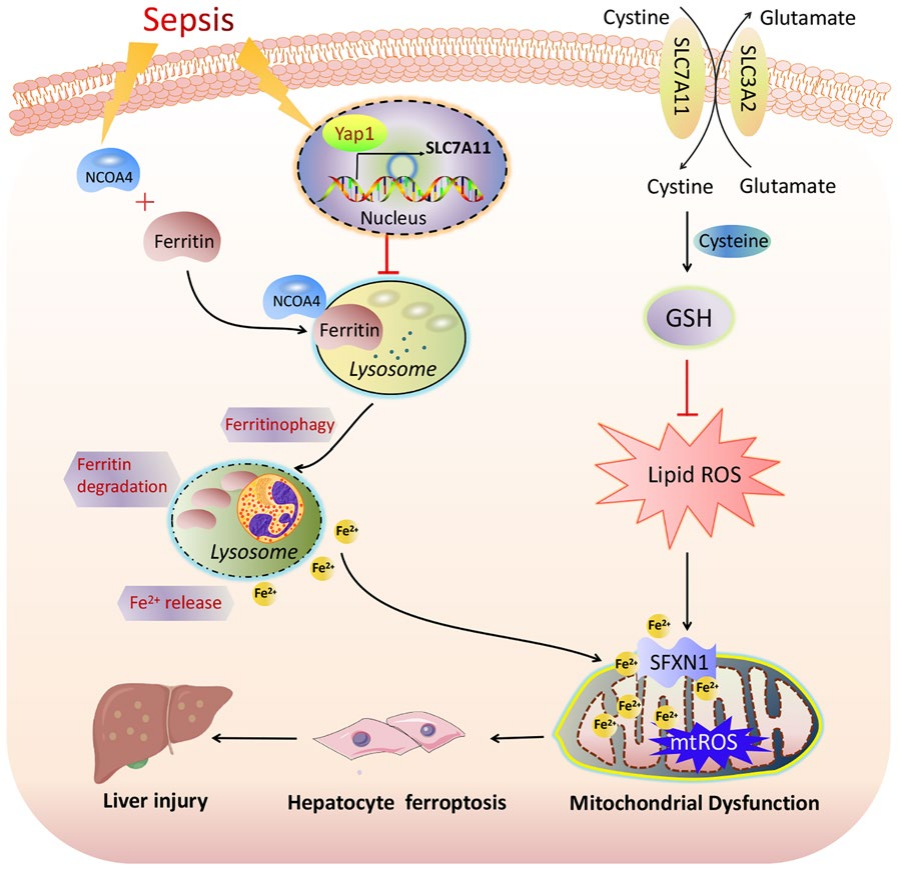
The mechanism illustration on the involvement of YAP1 in ferritinophagy and ferroptosis in sepsis-induced liver injury. YAP1 inhibits ferritin degradation into autophagosomes via disrupting NCOA4–FTH1 interaction, preventing ferrous iron from being releasing into mitochondria, which subsequently restrains the accumulation of mitochondrial reactive oxygen species and lipid peroxidation, thus protects hepatocyte from ferroptosis. Therefore, YAP1 involves in sepsis-induced acute liver injury by regulating the process of ferritinophagy-mediated ferroptosis

In conclusion, our results demonstrate that YAP1 suppresses ferritinophagy- mediated ferroptosis via disruption of NCOA4–FTH1 reaction, thus attenuates sepsis-induced liver injury, which raises the possibility that YAP1 might be a potential therapeutic target of septic liver injury (Fig. 8).

## Supplementary Information

**Additional file 1.** Supplementary figures.

## Data Availability

All data generated or analyzed during this study are included in this article.

## Abbreviations

YAP1: Yes-associated protein1
CLP: Cecal ligation and puncture
LPS: Lipopolysaccharide
GPX4: Glutathione Peroxidase 4
SLC7A11: Cystine/glutamate antiporter solute carrier family 7 member 11
FTH1: Ferritin heavy chain 1
SFXN1: Sideroflexin 1
NCOA4: Nuclear receptor coactivator 4
ROS: Reactive oxygen species
MODS: Multiple organ dysfunction syndrome
FTL: Ferritin light chain
LATS1/2: Large tumor suppressor 1/2
ALT: Alanine aminotransferase
AST: Aspartate aminotransferase
TEM: Transmission electron microscopy
HE: Hematoxylin–eosin
IHC: Immunohistochemical
Fe^2+^: Ferrous iron
MDA: Malondialdehyde
GSH: Glutathione
