# Supplementary material for: YAP1 protects against septic liver injury via ferroptosis resistance
Source: Cell Biosci. 2022 Oct 1;12:163. doi: 10.1186/s13578-022-00902-7 (PMC9526934; doi:10.1186/s13578-022-00902-7)
Supplement: Supplementary file 1 — Additional file 1. Supplementary figures. [file 13578_2022_902_MOESM1_ESM.docx]

**Additional file 1**

**YAP1 protects against septic liver injury via ferroptosis resistance**

We investigated the ideal LPS concentration and treatment period in order to build an inflammatory model of LPS in LO2 cells. First, LPS was applied to LO2 cells at varying concentrations (0.5 μg/mL, 1 μg/mL, 2 μg/mL, and 5μg/mL) for 24 hours. The number of viable cells was measured using the CCK-8 kit (Sigma-Aldrich, 96992). The optimal LPS concentration was determined to be 1 μg/mL based on the results, which demonstrated that when the concentration of LPS was higher than 1 μg/mL compared to the control group, the number of cells was dramatically reduced (Fig. S1 A) . Second, the number of cells was determined after treating LO2 cells with 1 μg/mL LPS for 6, 12, 24, and 48 hours (Fig. S1 B). When 1μg/mL LPS treatment lasted 24 hours or longer, the results showed that the number of cells was dramatically reduced in comparison to the undamaged control group, which were chosen as the concentration and time for cells treatment.


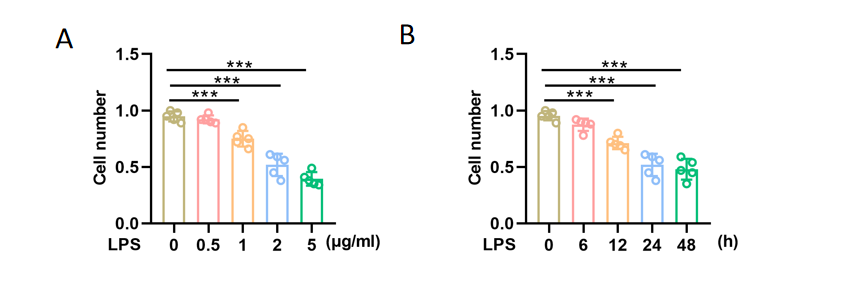


Fig. S1 Preliminary experiments on the concentration and timing selection of LPS stimulation. LO2 cells were stimulated with different doses (0.5 μg/mL, 1 μg/mL, 2 μg/mL, and 5μg/mL) of LPS for various durations (6, 12, 24, and 48) hours **(A, B)** using the CCK-8 kit. Data are expressed as mean ± SD of three replicates. n=5，***p＜0.001.

In order to determine whether LPS caused the ferroptosis of LO2 cells, we evaluated the effects of the ferroptosis inducer (erastin E7781, Sigma) and inhibitor (Fer-1, SML0583; Sigma) on LPS-treated cells. Four groups of LO2 cells were established: the control group (Con), the LPS group (LPS), the LPS + Ferrostatin-1 group (LPS+Fer-1), and the LPS + Erastin group (LPS+Era). The Era (20 μM) and Lip-1 (1 μM) pretreatments were given to each group 1 h before LPS stimulation. ROS and Fe^2+^ assays (Fig. S2 B, C) revealed that LPS significantly increased iron buildup and ROS production in LO2 cells. In LPS-treated lLO2 cells, Fer-1 could reduce iron accumulation and ROS generation, whereas Erastin had negative effects. Additionally, while erastin pretreatment demonstrated a more pronounced fall in GSH contents, but Fer-1 partially reversed the LPS-induced decrease in GSH (Fig. S2 A). These findings demonstrated that LPS stimulation of LO2 cells resulted in the activation of ferroptosis.


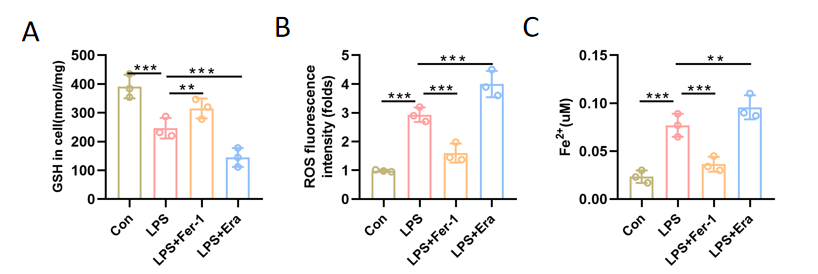


Fig. S2 LPS (1 μg/ml for 24 h) stimulation induced ferropotosis in LO2 cells. Intracellular levels of GSH **(A)**, ROS **(B**), and Fe^2+^ **(C)** in LO2 cells were measured by relevant kits. Data are expressed as mean ± SD of three replicates. n=3, *p＜0.05，**p＜0.01，***p＜0.001.

YAP is the primary downstream effector molecule of the Hippo signaling pathway and can be phosphorylated directly by LAST1/2. Phosphorylated YAP (p-YAP) binds to proteins in the cytoplasm before being ubiquitinated and degraded. When YAP enters the nucleus, it can regulate tissue regeneration and keep the balance between proliferation and apoptosis. The p-YAP1/YAP1 ratio in the liver dropped after LPS stimulation, indicating that YAP1 was activated. Moreover, YAP1 overexpression decreased the p-YAP1/YAP1 ratio, indicating that YAP1 was activated further after YAP1 OE (Fig. S3 A, B). Similarly, immunofluorescence studies revealed that LPS enhanced partial nuclei translocation of YAP1, while Yap1 overexpression in the presence of LPS considerably accelerated YAP1 nuclei translocation (Fig. S3 C).


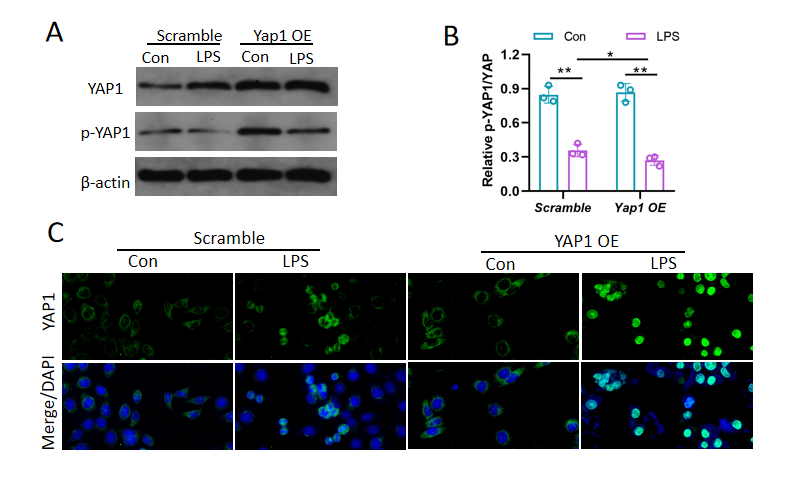


Fig. S3 The phosphorylation and the intracellular location of YAP1 in LO2 cells. **(A, B)** Western blot analysis of p-YAP1 and YAP1 protein in the scramble group and YAP1 overexpression group, n=3 **(C)** The fluorescence images of YAP1 (green) of nuclear translocation in indicated groups. Nuclei were stained by DAPI (blue). Data are expressed as mean ± SD of three replicates. *p＜0.05，**p＜0.01，***p＜0.001.
